# Effects of different surgical strategies and left ventricular remodelling on the outcomes of coronary artery bypass grafting in heart failure patients with reduced ejection fraction

**DOI:** 10.3389/fcvm.2024.1398700

**Published:** 2024-06-04

**Authors:** Jian Cao, Miao Yu, Yu Xiao, Ran Dong, Jiayang Wang

**Affiliations:** Department of Cardiac Surgery, Beijing Institute of Heart, Lung and Blood Vessel Diseases- Beijing Anzhen Hospital, Affiliated of Capital Medical University, Beijing, China

**Keywords:** on-pump coronary artery bypass grafting, off-pump coronary artery bypass grafting, heart failure and reduced ejection fraction, left ventricular remodelling, left ventrical end systolic volume index

## Abstract

**Background:**

Ischaemic heart failure with reduced ejection fraction (HFrEF) caused by coronary artery disease accounts for the largest proportion of heart failure cases with the worst prognosis. Coronary artery bypass grafting (CABG) is the most effective treatment for ischaemic HFrEF. On-pump and off-pump are the two surgical methods used for CABG. Whether patients with HFrEF should undergo on- or off-pump CABG is controversial in coronary heart disease surgery. The left ventricular end-systolic volume index (LVSEVI) is the gold standard for evaluating the severity of left ventricular remodelling; however, its effect on the perioperative risk and long-term survival rate of patients with HFrEF undergoing CABG remains unclear.

**Methods:**

This single centre prospective cohort analysis included 118 coronary heart disease patients with symptoms and signs of heart failure and a left ventricular ejection fraction (LVEF) of <40% who were enrolled consecutively from January 2019 to December 2023. Operative mortality, perioperative complications, and long-term survival were compared among patients treated with various LVESVIs and surgical methods. The primary outcomes were cardiac death, myocardial infarction, heart failure, stroke, and revascularization, (percutaneous coronary intervention or redo CABG) with a median follow-up of 38 ± 10 months.

**Results:**

The 30-day postoperative mortality of 118 patients was 6.8%. Patients in the off-pump group had significantly higher perioperative mortality than those in the on-pump group (12.5% vs. 3.8%, *p* = 0.03). In the off-pump group, a higher proportion of patients required perioperative mechanical assistance, such as intra-aortic artery balloon pump (IABP) or extracorporeal membrane oxygenation (ECMO), compared to those in the on-pump group (IABP: 75% vs. 47.4%, *p* = 0.004; ECMO: 22.5% vs. 1.3%, *p* = 0.000). Patients in the off-pump group were more likely to have postoperative atrial fibrillation (AF) (35% vs. 14.1%, *p* = 0.01). In the on-pump group, the incidence of postoperative AF (25% vs. 6.5%, *p* = 0.02) and IABP use (62.5% vs. 36.9%, *p* = 0.03) were significantly higher in patients with more severe left ventricular remodelling than in those with less severe left ventricular remodelling. In the off-pump group, patients with more severe left ventricular remodelling had higher ECMO usage (38.9% vs. 9.1%, *p* = 0.04), incidence of postoperative AF (61.1% vs. 13.6%, *p* = 0.02), and perioperative mortality (22.2%). Major adverse cardiac event (MACE)-free survival rate was significantly higher in the on-pump group than in the off-pump group, and there was no significant difference in MACE free survival rates between the two groups of patients with different degrees of left ventricular remodelling.

**Conclusion:**

On-pump bypass is a better surgical procedure for patients with ischaemic HFrEF, especially those with severe left ventricular remodelling. Left ventricular remodelling increases perioperative mortality but has no effect on long-term survival.

## 1 Introduction

Heart failure is a major health concern, and its incidence in developed countries is approximately 1%–2% (1). Depending on the ejection fraction, heart failure is classified as heart failure with reduced ejection fraction (HFrEF) or heart failure with preserved ejection fraction. According to previous research, 5.1 million adults in the United States are diagnosed with heart failure (2), with at least 50% of these patients having HFrEF defined as a left ventricular ejection fraction (LVEF) of less than 40% (3). The most common cause of HFrEF is ischaemic heart disease, accounting for approximately 60% of all causes (3). Coronary heart disease patients with heart failure have higher number of clinical comorbidities, rates of bleeding and re-myocardial infarction, and rates of undertreatment and mortality than those without heart failure (4). Coronary heart disease patients with HFrEF have a significantly worse prognosis than those without coronary heart disease patients and HFrEF (5, 6). Coronary artery bypass grafting (CABG) is the most effective treatment for patients with coronary heart disease and HFrEF. CABG is associated with lower rates of all-cause death, cardiovascular death, and rehospitalisation than those of medical therapy. Surgical methods for CABG mainly include on- and off-pump CABG. Off-pump CABG is a novel strategy in which the heart continues to beat while the graft is implanted. The technique was created to reduce the likelihood of adverse effects from cardiopulmonary bypass, including systemic inflammation and neuropsychological deficits. Off pump CABG aims to reduce issues, improve patient outcomes, and speed up postoperative recovery (7). Unlike the traditional on-pump technique, off-pump CABG surgery is performed without momentarily stopping the heart or connecting it to a heart-lung machine. Additionally, it allows the surgeon to operate on a beating heart, avoiding restricted or obstructed coronary arteries, thus allowing the heart to continue pumping blood. For patients who are more vulnerable to surgical complications, off-pump CABG offers a safer option by reducing the stress on the heart (8). However, off-pump CABG is associated with certain challenges. A high level of surgical knowledge and proficiency are required to perform the procedure on an active, beating heart. Accuracy is essential to guarantee successful grafting and prevent graft failure or insufficient revascularization (9). Large-scale randomised controlled trials are lacking to determine whether on-pump or off-pump CABG is better for HFrEF patients. Currently, the choice of surgical method for these patients is determined by the surgeon's experience and preferences.

## 2 Materials and methods

### 2.1 Patient population

Patients with symptoms and signs of heart failure and a LVEF of <40% were enrolled consecutively from January 2019 to December 2024. The exclusion criteria were cardiogenic shock, acute myocardial infarction within 3 months, and other concurrent surgeries. The patients were divided into on-pump and off-pump CABG groups according to the type of CABG performed. Each group was further divided into four subgroups according to the mean value of end systolic volume index (ESVI): on-pump small left ventricle group [left ventricular end-systolic volume index (LVESVI) <92 ml/m^2^], on-pump large left ventricle group (LVESVI ≧ 92 ml/m), on-pump small left ventricle group (LVESVI <92 ml/m), off-pump large left ventricle group (LVESVI ≧ 92 ml/m). All patients were treated with nitrates, aspirin, metoprolol, statins, and ACEI or ARB1 for more than 1 year.

### 2.2 Study design

This study was a prospective cohort study. The patients were stratified into on-pump and off-pump bypass groups based on the department's quality control committee deliberations to compare in-hospital mortality, perioperative complications (stroke, perioperative low cardiac output syndrome, renal failure, and perioperative myocardial infarction), and major adverse cardiac event (MACE)-free survival rate between on-pump and off-pump CABG patients with HFrEF.

### 2.3 Definition of outcomes

Perioperative death was defined as death during the hospitalisation period or within 30 days of surgery. Stroke was defined as transient or persistent postoperative neurological dysfunction confirmed through computed tomography or magnetic resonance imaging. Postoperative low cardiac output syndrome was defined as cardiac index <2.0 L/min·m^2^), and/or increased dose of inotropic drugs, and/or evidence of organ dysfunction (lactate level ≥2.5 mmol/L and/or urine output <0.5 ml/kg/h). Renal failure was defined as an increase in the serum creatinine level to twice the preoperative level or greater than 2.0 mg/dl or the need for dialysis. Periprocedural myocardial infarction was defined by at least two of the following criteria: persistent angina for more than 20 min, elevated myocardial enzymes (CK-MB level greater than one-tenth of the total CK level), new wall motion abnormalities, and ST-segment elevation in more than two consecutive leads on electrocardiography.

### 2.4 Measurement of left ventricular ejection fraction and end-systolic volume

The LVEF and LVESV were measured separately by two sonographers before the operation. If an EF of <40% was simultaneously identified by two sonographers, a third sonographer was consulted to confirm the diagnosis. LVESV was calculated as the average of the values measured by the two radiologists. The LVESVI was obtained by dividing the LVESV by the patient's body surface area.

### 2.5 CABG procedure

A Swan–Ganz catheter was implanted in all patients to measure cardiac output during anaesthesia. The surgical approach for off-pump bypass involves a median thoracic incision. The left internal mammary artery and great saphenous vein were used as the graft materials. The left internal mammary artery was anastomosed to the left anterior descending artery. The other diseased vessels were anastomosed to the great saphenous vein via side-to-side or end-to-side anastomosis. The ascending aorta and great saphenous vein were anastomosed with a proximal stapler (heartstring), and distal anastomosis was performed with a heart surface fixator. The flow in each graft was monitored through intraoperative transit time flow measurement. The surgical approach and graft materials for on-pump bypass were the same as those for off-pump bypass. Cold blood cardioplegia (4:1) was used with an initial perfusion volume of 20 ml/kg followed by half-volume reperfusion after 30 min for myocardial protection. The perfusion mode showed positive perfusion with graft perfusion, and the perfusion volume of each graft was 200 ml. The left internal mammary artery was anastomosed to the left anterior descending artery, and the other diseased vessels were anastomosed to the great saphenous vein in a sequential manner, side-to-side or end-to-side anastomosis.

### 2.6 Follow up

Patients were followed-up at 3 months, 6 months, 1 year, and annually thereafter. Echocardiography, electrocardiography, coronary computed tomography angiography, liver and kidney function tests, and cardiac function assessments were performed. The primary end points were all-cause mortality and MACE.

### 2.7 Statistical methods

The SPSS 25 software was used to analyse the data. The measurement data were tested for normality. Variables conforming to a normal distribution are expressed as means and standard deviations, and the comparison of means between the two groups was performed using the t-test. Variables that did not meet the normal distribution are described as median or interquartile range. Comparison of the medians between the two groups was performed using nonparametric statistics. The *χ*2 test or Fisher's exact test was used to compare the rates or ratios between the two groups. The survival rate was analysed using the Kaplan–Meier survival curve method. Statistical significance was tested by two-sided test, and *p* < 0.05 was considered as the criterion for significance.

## 3 Results

The mean LESVI of the entire cohort (118 patients) was 92 ml/m^2^. Forty patients who underwent off-pump bypass were assigned to the off-pump group, and 78 patients who underwent on-pump bypass were assigned to the on-pump group. There were no significant differences in the preoperative LVEF, LVESVI, or cardiac function between the on-pump and off-pump groups. The proportion of patients who had received previous interventional therapy was higher in the on-pump group than in the off-pump group (Table 1). No correlation was observed between the severity of left ventricular remodelling and ejection fraction or functional status in the on-pump group (Table 2). In contrast, in the off-pump group, more severe left ventricular remodelling was associated with lower ejection fraction and worse cardiac function. In addition, a higher proportion of patients with more severe left ventricular remodelling in the off-pump group had a previous myocardial infarction than those with less remodelling (Table 3).

**Table 1:** Baseline characteristics of patients in the off-pump group and the on-pump group.

Variables	On-pump (*N* = 78)	Off-pump (*N* = 40)	*P* value
Age	61 ± 6	59 ± 8	0.868
Diabetes	30 (38.5%)	16 (40.0%)	0.224
Hypertension	39 (50.0%)	19 (47.5%)	0.073
Hyperlipidaemia	33 (42.3%)	12 (30.0%)	0.722
CKD	2 (2.56%)	1 (2.50%)	0.956
PVD	10 (12.8%)	7 (17.5%)	0.992
Prior PCI	30 (38.5%)	15 (37.5%)	0.755
Prior MI	59 (75.6%)	24 (60.0%)	0.124
AF	3 (3.85%)	2 (5.0%)	0.070
Prior stroke	0	0	NS
NCAs	3.0 ± 0.52	3.2 ± 0.65	0.353
LM disease	7 (8.97%)	4 (10.0%)	0.856
NYHA			0.331
I	2 (2.56%)	1 (2.50%)	
II	20 (25.6%)	5 (12.5%)	
III	47 (60.3%)	30 (75.0%)	
IV	9 (11.5%)	4 (10.0%)	
BMI	26.3 ± 2.5	25.4 ± 2.7	0.433
LVEF (%)	32.5 ± 3.5	33.2 ± 3.1	0.735
LVEDVI (ml/m^2^)	119 ± 26	117 ± 32	0.612
LVESVI (ml/m^2^)	913 ± 21	95 ± 33	0.253
Syntax score	45.3 ± 4.1	48.2 ± 3.4	0.571

AF, atrial fibrillation; BMI, body mass index; CKD, chronic kidney disease; LM, left main disease; LVEF, left ventricular ejection fraction; LVEDVI, left ventricular end-diastolic volume index; LVESVI, left ventricular end-systolic volume index; NCAs, number of diseased coronary arteries; PVD, peripheral vascular disease.

**Table 2:** Baseline characteristics of the subgroup of patients in the on-pump group.

Variables	LVESVI <92 ml/m^2^ (*N* = 46)	LVESVI ≥92 ml/m^2^ (*N* = 32)	*P* value
Age	57 ± 6	60 ± 9	0.066
Diabetes	20 (43.5%)	10 (31.3%)	0.661
Hypertension	25 (54.3%)	14 (43.8%)	0.344
Hyperlipidaemia	18 (39.1%)	15 (46.9%)	0.711
CKD	1 (2.17%)	1 (3.13%)	0.554
PVD	5 (10.9%)	4 (12.5%)	0.641
Prior PCI	17 (36.9%)	13 (40.6%)	0.398
Prior MI	25 (54.3%)	34 (80.0%)	0.461
AF	2 (4.35%)	1 (3.13%)	0.737
Prior stroke	0	0	NS
NCAs	2.8 ± 0.7	3.1 ± 0.5	0.184
LM disease	5 (10.9%)	2 (6.25%)	0.394
NYHA			0.891
I	2 (4.35%)	0	
II	7 (15.2%)	13 (40.6%)	
III	25 (54.3%)	22 (68.8%)	
IV	4 (8.69%)	5 (15.6%)	
BMI	26.1 ± 3.0	24.9 ± 2.8	0.742
LVEF(%)	31.9 ± 3.5	32.7 ± 2.9	0.651
LVEDVI (ml/m^2^)	96 ± 14	142 ± 33	<0.010
LVESVI (ml/m^2^)	77 ± 17	120 ± 24	<0.010
Syntax score	44.1 ± 4.4	46.1 ± 3.9	0.662

AF, atrial fibrillation; BMI, body mass index; CKD, chronic kidney disease; LM, left main disease; LVEF, left ventricular ejection fraction; LVEDVI, left ventricular end-diastolic volume index; LVESVI, left ventricular end-systolic volume index; NCAs, number of diseased coronary arteries; PVD, peripheral vascular disease.

**Table 3:** Baseline characteristics of the subgroup of patients in the off-pump group.

Variables	LVESVI <92 ml/m^2^ (*N* = 22)	LVESVI ≥92 ml/m^2^ (*N* = 18)	*P* value
Age	55.5 ± 9.4	59.0 ± 8.8	0.612
Diabetes	11 (50.0%)	5 (27.8%)	0.721
Hypertension	9 (40.9%)	10 (55.6%)	0.502
Hyperlipidaemia	6 (27.3%)	6 (33.3%)	0.445
CKD	0	1 (5.56%)	NS
PVD	3 (13.6%)	4 (22.2%)	0.044
Prior PCI	9 (40.0%)	6 (33.3%)	0.217
Prior MI	9 (40.0%)	15 (83.3%)	0.033
AF	0	2 (11.1%)	NS
Prior stroke	0	0	NS
NCAs	3.2 ± 0.8	2.8 ± 0.7	0.091
LM disease	3 (13.6%)	1 (5.56%)	0.251
NYHA			0.021
I	1 (4.55%)	0	
II	4 (18.2%)	1 (5.56%)	
III	20 (90.9%)	10 (55.6%)	
IV	2 (9.09%)	2 (11.1%)	
BMI	25.7 ± 1.4	24.6 ± 1.9	0.277
LVEF (%)	35.5 ± 3.3	32.7 ± 3.3	0.012
LVEDVI (ml/m^2^)	97 ± 20	149 ± 32	<0.010
LVESVI (ml/m^2^)	68 ± 15	127 ± 26	<0.010
Syntax score	45.5 ± 3.8	48.9 ± 2.9	0.511

AF, atrial fibrillation; BMI, body mass index; CKD, chronic kidney disease; LM, left main disease; LVEF, left ventricular ejection fraction; LVEDVI, left ventricular end-diastolic volume index; LVESVI, left ventricular end-systolic volume index; NCAs, number of diseased coronary arteries; PVD, peripheral vascular disease.

The perioperative mortality rate of the entire cohort was 6.8%. Patients in the off-pump group had a significantly higher perioperative mortality than those in the on-pump group (12.5% vs. 3.8%, *p* = 0.03). A higher number of patients in the off-pump group required perioperative mechanical assistance such as intra-aortic artery balloon pump (IABP) or extracorporeal membrane oxygenation (ECMO) (IABP: 75% vs. 47.4%, *p* = 0.004; ECMO: 22.5% vs. 1.3%, *p* = 0.000) than those in the on-pump group. Patients in the off-pump group were more likely to have postoperative atrial fibrillation (AF) (35% vs. 14.1%, *p* = 0.01) (Table 4). According to the mean LVESVI value of 92 ml/m^2^, the incidence of postoperative AF (25% vs. 6.5%, *p* = 0.02) and IABP usage (62.5% vs. 36.9%, *p* = 0.03) in on-pump group patients with more severe left ventricular remodelling were significantly higher than those in patients with less severe left ventricular remodelling. Mortality rate was also higher, although not significantly (6.3% vs. 2.2%, *p* = 0.56) (Table 5). In the off-pump group, patients with more severe left ventricular remodelling had a higher rate of ECMO use (38.9% vs. 9.1%, *p* = 0.04), incidence of postoperative AF (61.1% vs. 13.6%, *p* = 0.02), and perioperative mortality rate (22.2%) than those with less severe left ventricular remodelling, and this group had the highest surgical risk (Table 6).

**Table 4:** Surgical data of patients in the off-pump and on-pump groups.

Variables	On-pump (*N* = 78)	Off-pump (*N* = 40)	*P* value
Operative time (min)	261 ± 48	236 ± 55	0.271
Number of grafts	3.2 ± 0.9	3.1 ± 0.8	0.354
Total SVGs	32 (41.0%)	15 (37.5%)	0.715
RBC transfusion (U)	2.0 ± 1.1	0.8 ± 0.4	0.083
ICU stay (h)	83 ± 65	90 ± 38	0.434
Duration of MV (h)	48 ± 33	58 ± 38	0.101
Complications			
Death	3 (3.8%)	5 (12.5%)	0.034
Cardiac arrest	2 (2.6%)	4 (10.0%)	0.082
Stroke	0	0	NS
PMI	2 (2.6%)	2 (5.0%)	0.491
AF	11 (14.1%)	14 (35%)	0.011
Pulmonary infection	11 (14.1%)	9 (22.5%)	0.255
Tracheotomy	1 (1.3%)	0	0.473
CRRT	5 (6.4%)	6 (15.0%)	0.133
Secondary thoracotomy	0	2 (5.0%)	0.116
IABP	37 (47.4%)	30 (75.0%)	0.004
IABP time (h)	115 ± 89	113 ± 51	0.092
ECMO	1 (1.3%)	9 (22.5%)	<0.010

AF, atrial fibrillation; CRRT, continuous renal replacement therapy; ECMO, extracorporeal membrane oxygenation; IABP, intra-aortic artery balloon pump; PMI, postoperative myocardial infarction; RBC, red blood cell; SVG, saphenous vein graft.

**Table 5:** Surgical data of patients in the on-pump group.

Variables	LVESVI <92 ml/m^2^ (*N* = 46)	LVESVI ≥92 ml/m^2^ (*N* = 32)	*P* value
Operative time (min)	241 ± 57	276 ± 44	0.340
Number of grafts	3.3 ± 0.9	3.1 ± 0.8	0.322
Total SVGs	18 (39.1%)	14 (43.8%)	0.686
RBC transfusion (U)	1.0 ± 1.5	1.2 ± 0.9	0.590
ICU stay (h)	73 ± 52	96 ± 41	0.133
Duration of MV (h)	42 ± 38	55 ± 45	0.233
Complications			
Death	1 (2.2%)	2 (6.3%)	0.561
Cardiac arrest	0	2 (6.3%)	0.512
Stroke	0	0	NS
PMI	1 (2.2%)	1 (3.1%)	0.794
AF	3 (6.5%)	8 (25.0%)	0.024
Pulmonary infection	6 (13.0%)	5 (15.6%)	0.751
Tracheotomy	0	1 (3.1%)	0.417
CRRT	2 (4.3%)	3 (9.4%)	0.393
Secondary thoracotomy	0	0	NS
IABP	17 (36.9%)	20 (62.5%)	0.031
IABP time (h)	96 ± 50	130 ± 108	0.143
ECMO	1 (2.2%)	0	1.000

AF, atrial fibrillation; CRRT, continuous renal replacement therapy; ECMO, extracorporeal membrane oxygenation; IABP, intra-aortic artery balloon pump; PMI, postoperative myocardial infarction; RBC, red blood cell; SVG, saphenous vein graft.

**Table 6:** Surgical data of patients in the off-pump group.

Variables	LVESVI <92 ml/m^2^ (*N* = 22)	LVESVI ≥92 ml/m^2^ (*N* = 18)	*P* value
Operative time (min)	227 ± 64	277 ± 55	0.211
Number of grafts	3.2 ± 0.5	3.0 ± 0.8	0.952
Total SVGs	8 (36.4%)	7 (38.9%)	0.872
RBC transfusion (U)	0.5 ± 0.8	1.5 ± 1.4	0.127
ICU stay (h)	81 ± 47	97 ± 55	0.375
Duration of MV (h)	52 ± 30	66 ± 33	0.528
Complications			
Death	1 (4.5%)	4 (22.2%)	<0.010
Cardiac arrest	2 (9.1%)	2 (11.1%)	1.000
Stroke	0	0	NS
PMI	2 (9.1%)	0	0.493
AF	3 (13.6%)	11 (61.1%)	0.031
Pulmonary infection	5 (22.7%)	4 (22.2%)	0.972
Tracheotomy	0	0	NS
CRRT	3 (13.6%)	3 (16.7%)	0.794
Secondary thoracotomy	1	1	0.892
IABP	18 (81.8%)	12 (66.7%)	0.277
IABP time (h)	110 ± 46	118 ± 62	0.686
ECMO	2 (9.1%)	7 (38.9%)	0.043

AF, atrial fibrillation; CRRT, continuous renal replacement therapy; ECMO, extracorporeal membrane oxygenation; IABP, intra-aortic artery balloon pump; PMI, postoperative myocardial infarction; RBC, red blood cell; SVG, saphenous vein graft.

**Figure 1:**
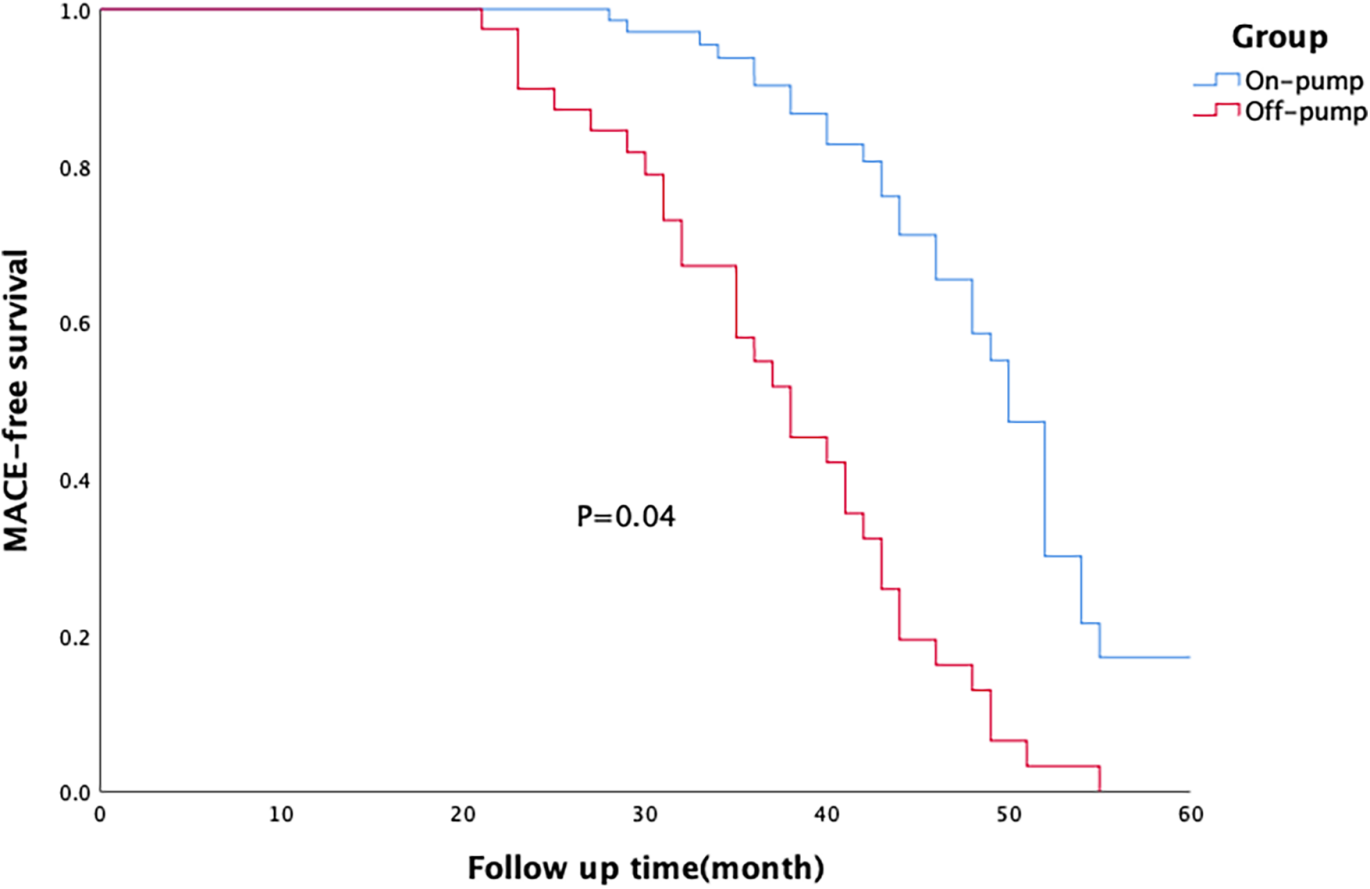
Survival of patients in the on-pump and off-pump coronary artery bypass grafting (CABG) groups. The major adverse cardiac event (MACE)-free survival rate of patients in on-pump CABG group was significantly higher than that of patients in on-pump CABG group.

**Figure 2:**
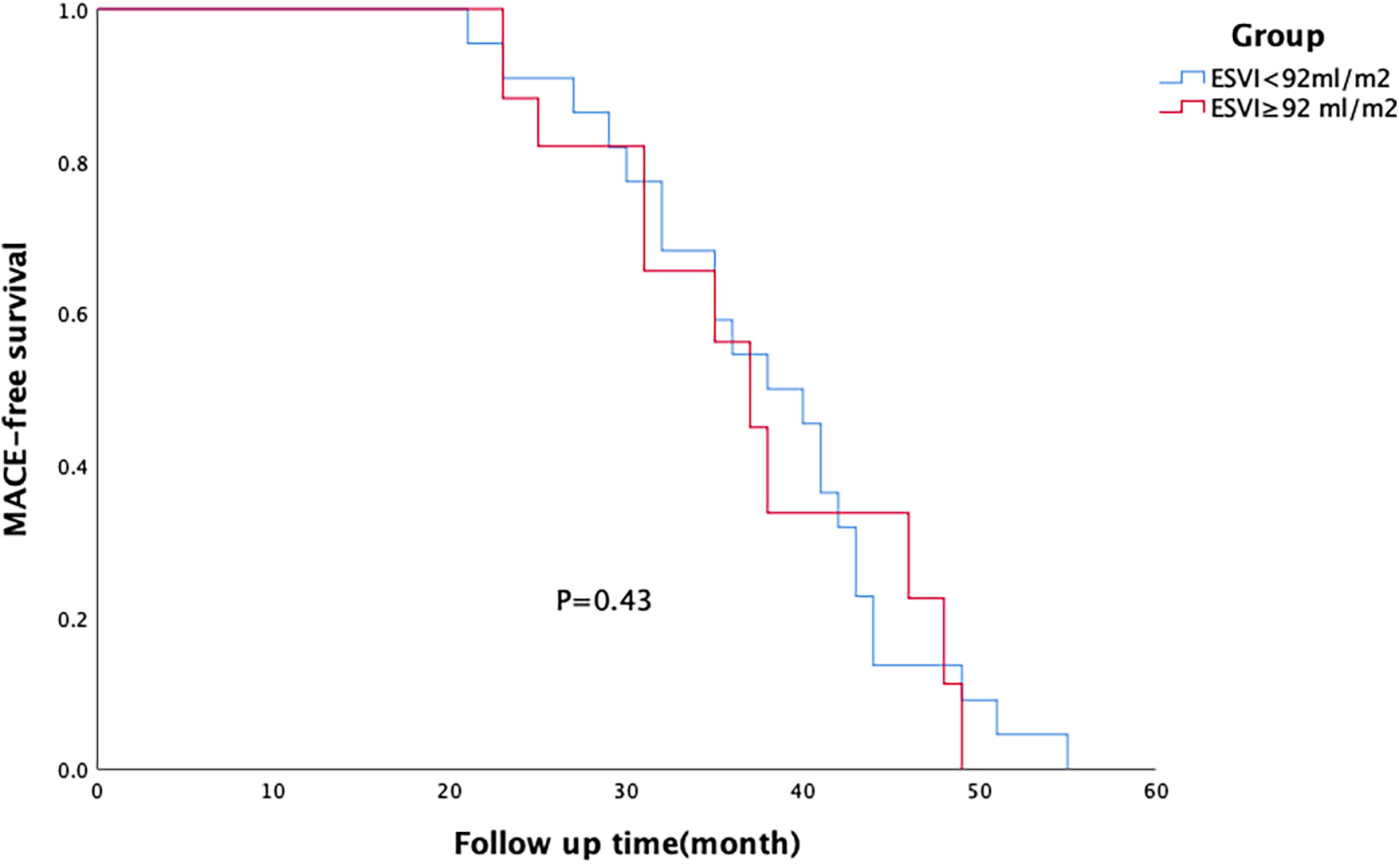
Survival of patients in off-pump coronary artery bypass grafting (CABG) group. There was no difference in the major adverse cardiac event (MACE)-free survival rate between patients with different degrees of left ventricular remodelling within each group.

**Figure 3:**
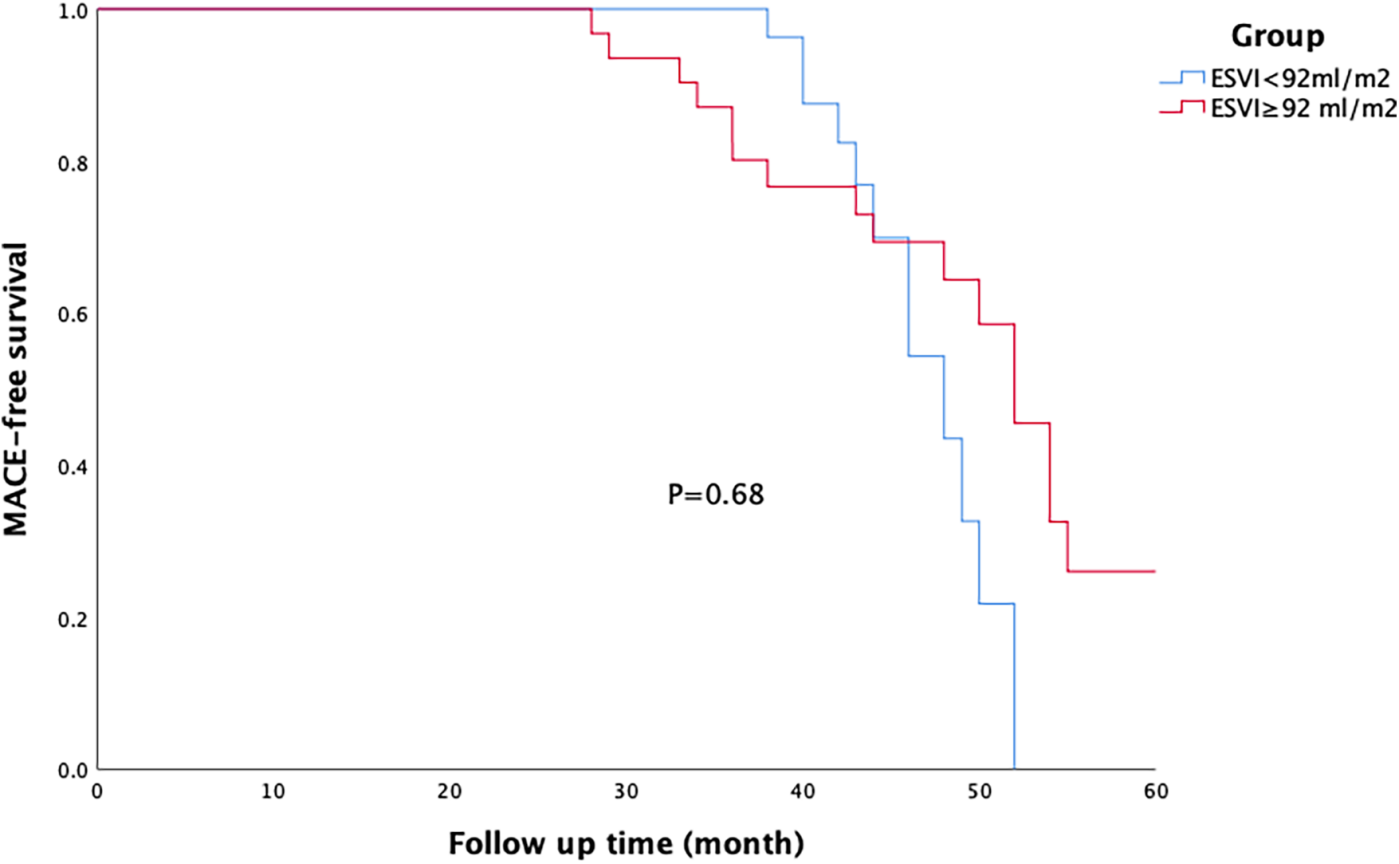
Survival of patients in the on-pump coronary artery bypass grafting (CABG) group. There was no difference in the major adverse cardiac event (MACE)-free survival rate between patients with different degrees of left ventricular remodelling within each group.

All patients were followed up for an average period of 38 ± 10 months. MACE-free survival rate was significantly higher in the on-pump group than in the off-pump group (Figure 1), and there was no difference in this rate between the two groups of patients with different degrees of left ventricular remodelling (Figures 2, 3). The prognosis of patients in the on-pump group was better, and the severity of left ventricular remodelling had no effect on the long-term prognosis.

## 4 Discussion

We conducted a prospective single-centre study to analyse the data of 118 patients with HFrEF who underwent CABG. The patients were divided into two groups depending on the intraoperative surgical method: on-pump CABG and off-pump CABG.Within each group, patients were further stratified into two subgroups based on their LVESVI. The perioperative and long-term survival rates of coronary artery disease patients with heart failure and reduced EF after CABG were analysed by combining different surgical methods and LVESVI. Our results indicated that for patients with ischaemic HFrEF, on-pump CABG not only had a significantly lower perioperative mortality rate than that of off-pump CABG but also had a significantly better long-term survival rate than that of off-pump CABG. Off-pump CABG has a very high perioperative risk (22.2%), especially in patients with significant left ventricular enlargement. Severe left ventricular remodelling significantly increases the perioperative mortality rate of off-pump CABG patients but has no effect on their long-term survival.

Over the past decade, the comparative efficacy of off-pump and on-pump CABG has been controversial. Numerous randomised controlled trials have compared these two surgical methods. The CORONARY and GOPCABE trials demonstrated similar outcomes in terms of mortality, stroke, myocardial infarction, repeat revascularization, and composite endpoints at 1 and 5 years (10–13). However, the ROOBY trial revealed that the off-pump group had a worse composite outcome at 1 year (10), as well as lower 5-year survival rate and event-free survival rate than those of on-pump CABG (14). More recently, the ROOBY trial reported 10-year follow-up results and did not find any advantage of off-pump CABG in terms of 10-year mortality or revascularization endpoints (15). Therefore, it remains uncertain whether on- or off-pump CABG impacts survival. Consistent with the results of the ROOBY trial, our study also showed increased mortality in patients undergoing OPCAB which was particularly evident in those with severe left ventricular remodelling. It is important to note that criticism has been directed towards the design of the ROOBY trial owing to its inclusion of a population of patients at lower risk and participating surgeons with limited off-pump experience (16). The enrolled patients were high-risk surgical candidates with severely impaired LVEF. All procedures were performed by a single surgeon with extensive experience, who has conducted over 400 off-pump bypass surgeries annually, thus partially mitigating the limitations of the ROOBY trial.

Severe left ventricular dysfunction is an independent risk factor for early and long-term mortality after CABG (17–20), with an in-hospital mortality rate of 6.8%, as found in our study. The STICH trial reported a 30-day mortality rate of 5.1%, while the Society of Thoracic Surgeons (STS) national database indicated an in-hospital mortality rate of 3.1% after ONCAG and OPCAB. In the Japanese Adult Cardiovascular Surgery Database, postoperative mortality rates were 6.1% for OCAB and 3.3% for OPCAB, respectively (21, 22); these findings are consistent with our results. Previous studies have demonstrated that compared with on-pump CABG, off-pump CABG reduces operative mortality and early morbidity (22–24). According to the STS National Database, off-pump CABG is associated with a significantly decreased risk of early morbidity and mortality in patients with EF <0.30 (22). However, this advantage has not been sustained over the medium-to-long term (24, 25). Zhou et al. analysed ischaemic cardiomyopathy and the outcomes of on- and off-pump CABG in the STICH cohort and concluded that off-pump CABG could be performed with comparable 30-day mortality and long-term survival rates and appears to have a lower incidence of perioperative morbidities (26); the findings of these studies are inconsistent with our conclusions. Our analysis showed that the mortality rate was 2.2% after on-pump CABG and 4.5% after off-pump CABG in patients with lower ESVI and that both surgical methods were safe in these patients. There was no significant difference in LVESVI between the on-pump CABG group and the off-pump CABG group (91 ± 29 vs. 94 ± 36, *P* = 0.15), but the on-pump CABG group had a lower operative mortality than the off-pump CABG group (3.8% vs. 12.5%, *P* = 0.03). The incidence of postoperative AF was lower (14.1% vs. 35.0%, *P* = 0.01), and the use of perioperative mechanical assistance was also lower (IABP: 75% vs. 47.4%, *p* = 0.004; ECMO: 22.5% vs. 1.3%, *p* = 0.000), suggesting that on-pump CABG is a safer procedure. Although the perioperative mortality of patients with mild left ventricular remodelling in the off-pump group was 4.5%, which was comparable to that in the off-pump group, the perioperative mortality of patients with severe left ventricular remodelling was as high as 22.2%, and the rate of postoperative mechanical assistance was extremely high, which led to the poorer overall prognosis of patients in the off-pump group than that in the off-pump group. LVESVI positively correlated with mortality in patients undergoing the same surgical procedure. LVESVI is another important factor affecting surgical mortality and complications. We believe several reasons contribute to this situation; patients with severe left ventricular remodelling have a large degree of intraoperative heart displacement, and the haemodynamics are more likely to be unstable, which reduces myocardial perfusion and leads to cardiac function damage. To maintain haemodynamic stability during surgery, large doses pf vasoactive drugs are used, leading to peripheral vascular contraction and damage to the surrounding organs. Therefore, for patients with coronary heart disease combined with HFrEF, the choice of surgical method should fully consider the severity of left ventricular remodelling. Regarding the experience of surgeons and the number of anastomoses, as well as incomplete revascularization, we think these may not the main reasons for the poor prognosis of these patients. Our centre completes more than 1,400 cases of coronary artery bypass surgery annually, with over 90% being off-pump bypass surgeries. Surgeons perform more than 400 off-pump bypass surgeries annually, indicating extensive experience in this technique. There was no significant difference in the number of graft vessels between the on-pump and off-pump bypass groups (3.2 ± 0.9 vs. 3.1 ± 0.8, *P* = 0.35), and the degree of revascularization in the two groups was comparable.

Regarding long-term prognosis, a series of previous studies have suggested that improvement in cardiac function after bypass surgery occurs in patients with less left ventricular remodelling. However, there was no significant improvement in cardiac function in patients with severe left ventricular remodelling after bypass surgery. Yamaguchi et al. (27) followed up 20 bypass patients with LVEF <30% and found that patients with LVESVI <100 ml/m^2^ had significant postoperative cardiac function improvement; however, patients with LVESVI ≧ 100 ml/m^2^ showed no significant difference compared to their preoperative condition. Similarly, Bax et al. (28) found significant postoperative improvement in cardiac function among patients with smaller ESV during follow-up after bypass surgery. This improvement was observed in patients with an average LVEF of 29%. However, none of these studies compared the long-term survival rates. In this study, LVESVI was used as a grouping variable to compare the long-term event-free survival rates of patients undergoing the same surgical procedure which compensated for the shortcomings of the above studies. During a mean follow-up of 38 ± 10 months, no correlation was found between LVESVI and event-free survival in patients undergoing the same procedure, suggesting that the severity of left ventricular remodelling is not associated with long-term survival. There appears to be no correlation between left ventricular remodelling, improvement in left ventricular function after surgery, and long-term survival. However, whether and how these three factors interact need to be confirmed in future studies.

## 5 Conclusion

This study results suggests that on-pump bypass is a safer surgical procedure for patients with ischaemic HFrEF, especially those with severe left ventricular remodelling. Left ventricular remodelling increases perioperative mortality but has no effect on long-term survival.

## 6 Limitation of the study

Owing to the differences in surgical methods selected by different surgeons, it is not feasible for all operations to be performed by a single surgeon. This variation may introduce bias attributable to the surgeon. In addition, the sample size was small, and further studies are required to draw more reliable conclusions. Limited by the sample size, we used the mean LVESVI (92 ml/m^2^) as the cutoff value for classifying the severity of left ventricular remodelling in this study. We concluded that left ventricular remodelling had no effect on the long-term survival rate of the patients; however, this conclusion is not necessarily accurate. With an increased sample size, we plan to adopt a more scientific grounded classification of the severity of left ventricular remodelling (< 60 ml/m^2^, 60–90 ml/m^2^, ≧90 ml/m^2^) more scientifically in the future. This will allow for a more accurate evaluation of the effect of left ventricular remodelling on perioperative risk and long-term survival rates.

## Data Availability

The raw data supporting the conclusions of this article will be made available by the authors, without undue reservation.

## References

[1] Go AS Mozaffarian D Roger VL Benjamin EJ Berry JD Blaha MJ Heart disease and stroke statistics—2014 update: a report from the American Heart Association. Circulation. (2014) 129(3):e28–292. 10.1161/01.cir.0000441139.02102.80 24352519 PMC5408159

[2] Owan TE Hodge DO Herges RM Jacobsen SJ Roger VL Redfield MM. Trends in prevalence and outcome of heart failure with preserved ejection fraction. N Engl J Med. (2006) 355:251–9. 10.1056/NEJMoa052256 16855265

[3] Yancy CW Jessup M Bozkurt B Butler J Casey DE Jr Drazner MH. 2013 ACCF/AHA guideline for the management of heart failure: a report of the American college of cardiology foundation/American heart association task force on practice guidelines. J Am Coll Cardiol. (2013) 62:e147–239. 10.1016/j.jacc.2013.05.019 23747642

[4] Felker GM Shaw LK O’Connor CM. A standardized definition of ischemic cardiomyopathy for use in clinical research. J Am Coll Cardiol. (2002) 39:210–8. 10.1016/S0735-1097(01)01738-7 11788209

[5] McMurray JJ Adamopoulos S Anker SD Auricchio A Böhm M Dickstein K, et al. ESC Guidelines for the diagnosis and treatment of acute and chronic heart failure 2012: the TaskForce for the diagnosis and treatment of acute and chronic heart failure 2012 of the European Society of Cardiology. Developed in collaboration with the heart failure association (HFA) of the ESC. Eur Heart J. (2012) 33:1787–847. 10.1093/eurheartj/ehs104 22611136

[6] Manley JC King JF Zeft HJ. The “bad” left ventricle: results of coronary surgery and effect on late survival. J Thorac Cardiovasc Surg. (1976) 72:841–8. 10.1016/S0022-5223(19)40001-9 994534

[7] Bittner HB Savitt MA. Off-pump coronary artery bypass grafting: excellent results in a group of selected high-risk patients. Ann Thorac Surg. (2002) 74:115–8. 10.1016/s0003-4975(02)03646-9 12118740

[8] Vallely M Bannon P Kritharides L. The systemic inflammatory response syndrome and off-pump cardiac surgery. Heart Surg. (2001) 4:S7–S13. 11178301

[9] Yokoyama T Baumgartner FJ Gheissari A Capouya ER Panagiotides GP Declusin RJ. Off-pump versus on-pump coronary bypass in high-risk subgroups. Ann Thorac Surg. (2000) 70:1546–50. 10.1016/S0003-4975(00)01922-6 11093485

[10] Shroyer A Grover F Hattler B Carr BM Collins JF Almassi GH On-pump versus off-pump coronary artery bypass surgery. N Eng J Med. (2009) 361:1827–37. 10.1056/NEJMoa0902905 19890125

[11] Diegeler A Börgermann J Kappert U Breuer M Böning A Ursulescu A Off-pump versus on-pump coronary-artery bypass grafting in elderly patients. N Eng J Med. (2013) 368:1189–98. 10.1056/NEJMoa1211666 23477657

[12] Lamy A Devereaux P Prabhakaran D Taggart DP Hu S Straka Z Five-year outcomes after off-pump or on-pump coronary-artery bypass grafting. N Eng J Med. (2016) 375:2359–68. 10.1056/NEJMoa1601564 27771985

[13] Diegeler A Börgermann J Kappert U Hilker M Doenst T Böning A Five-year outcome after off-pump or on-pump coronary artery bypass grafting in elderly patients. Circulation. (2019) 139:1865–71. 10.1161/CIRCULATIONAHA.118.035857 30732456

[14] Shroyer A Hattler B Wagner T. Five-year outcomes after on-pump and off-pump coronary-artery bypass. N Eng J Med. (2017) 377:623–32. 10.1056/NEJMoa1614341 28813218

[15] Quin J Wagner T Hattler B Carr BM Collins J Almassi GH Ten-year outcomes of off-pump versus on-pump coronary artery bypass grafting in the department of veterans affairs: a randomized clinical trial. JAMA Surg. (2022) 157:303–10. 10.1001/jamasurg.2021.7578 35171210 PMC8851363

[16] Head SJ Milojevic M Taggart DP Puskas JD. Current practice of state-of-the art surgical coronary revascularization. Circulation. (2017) 136:1331–45. 10.1161/CIRCULATIONAHA.116.022572 28972063

[17] Topkara VK Cheema FH Kesavaramanujam S Mercando ML Cheema AF Namerow PB Coronary artery bypass grafting in patients with low ejection fraction. Circulation. (2005) 112(Suppl I):I344–350. 16159844 10.1161/CIRCULATIONAHA.104.526277

[18] Appoo J Norris C Merali S Graham MM Koshal A Knudtson ML Long-term outcomes of isolated coronary artery bypass surgery in patients with severe left ventricular dysfunction. Circulation. (2004) 110(Suppl 2):II133–217. 10.1161/01.CIR.0000138345.69540.ed 15364831

[19] Filsoufi F Rahmanian PB Castillo JG Chikwe J Kini AS Adams DH. Results and predictors of early and late outcomes of coronary artery bypass grafting in patients with severely depressed left ventricular function. Ann Thorac Surg. (2007) 84:808–16. 10.1016/j.athoracsur.2007.04.117 17720380

[20] Nardi P Pellegrino A Scafuri A Colella D Bassano C Polisca P Long-term outcome of coronary artery bypass grafting in patients with left ventricular dysfunction. Ann Thorac Surg. (2009) 87:1401–7. 10.1016/j.athoracsur.2009.02.062 19379873

[21] Wrobel K Stevens SR Jones RH Selzman CH Lamy A Beaver TM Influence of baseline characteristics,operative conduct, and postoperative course on 30-day outcomes of coronary artery bypass grafting among patients with left ventricular dysfunction: results from the surgical treatment for ischemic heart failure (STICH) trial. Circulation. (2015) 132:720–30. 10.1161/CIRCULATIONAHA.114.014932 26304663 PMC4551105

[22] Keeling WB Williams ML Slaughter MS Zhao Y Puskas JD. Off-pump and on-pump coronary revascularization in patients with low ejection fraction: a report from the society of thoracic surgeons national database. Ann Thorac Surg. (2013) 96:83–9. 10.1016/j.athoracsur.2013.03.098 23743061

[23] Al-Ruzzeh S Nakamura K Athanasiou T Modine T George S Yacoub M Does off-pump coronary artery bypass (OPCAB) surgery improve the outcome in high-risk patients?: a comparative study of 1398 high-risk patients. Eur J Cardiothorac Surg. (2003) 23:50–5. 10.1016/S1010-7940(02)00654-1 12493504

[24] Jarral OA Saso S Athanasiou T. Off-pump coronary artery bypass in patients with left ventricular dysfunction: a meta-analysis. Ann Thorac Surg. (2011) 92:1686–94. 10.1016/j.athoracsur.2011.06.006 21937020

[25] Jarral OA Saso S Athanasiou T. Does off-pump coronary artery bypass surgery have a beneficial effect on mortality in patients with left ventricular dysfunction? Interact Cardiovasc Thorac Surg. (2012) 14:856–64. 10.1093/icvts/ivs067 22422876 PMC3352731

[26] Zhou Z Liang M Zhuang X Liu M Fu G Liu Q Long-term outcomes after on-pump vs off-pump coronary artery bypass grafting for ischemic cardiomyopathy. Ann Thorac Surg. (2023) 115(6):1421–8. 10.1016/j.athoracsur.2021.12.063 35085524

[27] Yamaguchi A Ino T Adachi H Mizuhara A Murata S Kamio H. Left ventricular end-systolic volume index in patients with ischemic cardiomyopathy predicts postoperative ventricular function. Ann Thorac Surg. (1995) 60:1059–62. 10.1016/0003-4975(95)00488-7 7574948

[28] Bax JJ Schinkel AFL Boersma E Elhendy A Rizzello V Maat A Extensive left ventricular remodelling does not allow viable myocardium to improve in left ventricular ejection fraction after revascularization and is associated with worse long-term prognosis. Circulation. (2004) 110:18–22. 10.1161/01.CIR.0000138195.33452.b0 15364832

